# Multiscale cortical morphometry reveals pronounced regional and scale-dependent variations across the lifespan

**DOI:** 10.1093/cercor/bhaf154

**Published:** 2025-06-23

**Authors:** Karoline Leiberg, Timo Blattner, Bethany Little, Victor B B Mello, Fernanda H P de Moraes, Christian Rummel, Peter N Taylor, Bruno Mota, Yujiang Wang

**Affiliations:** CNNP Lab (www.cnnp-lab.com), Interdisciplinary Computing and Complex BioSystems Group, School of Computing, Newcastle University, 1 Science Square, NE4 5TG, Newcastle upon Tyne, United Kingdom; Support Center for Advanced Neuroimaging (SCAN), University Institute of Diagnostic and Interventional Neuroradiology, Inselspital, Bern University Hospital, Freiburgstrasse, CH-3010, Bern, Switzerland; CNNP Lab (www.cnnp-lab.com), Interdisciplinary Computing and Complex BioSystems Group, School of Computing, Newcastle University, 1 Science Square, NE4 5TG, Newcastle upon Tyne, United Kingdom; Support Center for Advanced Neuroimaging (SCAN), University Institute of Diagnostic and Interventional Neuroradiology, Inselspital, Bern University Hospital, Freiburgstrasse, CH-3010, Bern, Switzerland; metaBIO Lab, Instituto de Física, Universidade Federal do Rio de Janeiro (UFRJ), Av. Athos da Silveira Ramos 149, 21941-909, Rio de Janeiro, Brazil; Brain Connectivity Unit, D’Or Institute of Research and Education (IDOR), Rua Diniz Cordeiro 30, 22281-100, Rio de Janeiro, Brazil; Support Center for Advanced Neuroimaging (SCAN), University Institute of Diagnostic and Interventional Neuroradiology, Inselspital, Bern University Hospital, Freiburgstrasse, CH-3010, Bern, Switzerland; CNNP Lab (www.cnnp-lab.com), Interdisciplinary Computing and Complex BioSystems Group, School of Computing, Newcastle University, 1 Science Square, NE4 5TG, Newcastle upon Tyne, United Kingdom; Faculty of Medical Sciences, Newcastle University, Framlington Place, NE2 4HH, Newcastle upon Tyne, United Kingdom; UCL Queen Square Institute of Neurology, Queen Square, WC1N 3BG, London, United Kingdom; metaBIO Lab, Instituto de Física, Universidade Federal do Rio de Janeiro (UFRJ), Av. Athos da Silveira Ramos 149, 21941-909, Rio de Janeiro, Brazil; CNNP Lab (www.cnnp-lab.com), Interdisciplinary Computing and Complex BioSystems Group, School of Computing, Newcastle University, 1 Science Square, NE4 5TG, Newcastle upon Tyne, United Kingdom; Faculty of Medical Sciences, Newcastle University, Framlington Place, NE2 4HH, Newcastle upon Tyne, United Kingdom; UCL Queen Square Institute of Neurology, Queen Square, WC1N 3BG, London, United Kingdom

**Keywords:** aging, length scale, morphology, structural MRI

## Abstract

Characterizing changes in cortical morphology across the lifespan is fundamental for both research and clinical applications. Most studies report a monotonic decrease in commonly used morphometrics, such as cortical thickness and volume, with only subtle regional variations in the rate of decline. However, these findings are limited to a single length scale. Here, we delineate changes across the lifespan in multiscale morphometrics. We applied multiscale morphometric analysis to structural MRI from subjects aged 6 to 88 years from Nathan Kline Institute Rockland Sample (*n* = 833) and Cambridge Centre for Ageing and Neuroscience (*n* = 641), and derived population-level lifespan trajectories at multiple length scales. Lifespan trajectories show diverging and even opposing trends at different spatial scales. Larger scales (1.86 mm) displayed the strongest changes across the lifespan (up to 60%) when considering entire cortical hemispheres. Lobal variations also became more pronounced in scales over 0.7 mm. In a proof-of-principle brain age prediction context, multiscale morphometrics provided additional predictive value, boosting the adjusted $R^{2}$ of the model from 0.35 to 0.7. Our study provides a comprehensive multiscale description of cortical morphology across the lifespan, forming foundations for normative models to identify multiscale morphological abnormalities. Our results reveal the complementary information contained in different spatial scales, suggesting that morphometrics should be considered at multiple length scales.

## Introduction

Cortical morphology undergoes significant changes across the lifespan, noticeable even visually. Models of such effects are important for understanding the biological processes that underpin the lifespan, as well as for clinical applications to identify morphological abnormalities. Previous work describing population trends associated with healthy aging was predominantly performed in the well-established morphological metrics of cortical thickness, surface area, and volume, and studies usually consider each metric individually. Volume decreases with age have been reported mainly in frontal, but also in parietal and temporal regions (Good 2001; Resnick et al. 2003; Salat 2004; Storsve 2014; Bethlehem 2022). Gray matter volume loss is around 2.4 cm$^{3}$ per year in adults, with a faster decrease seen in advanced age (Resnick et al. 2003). Regional differences comprise mostly of subtle variations in the monotonic decrease; eg a faster decrease is seen in earlier decades oradvancing age (Storsve 2014; Frangou 2021). Decreases of cortical thickness with age have been described frequently (Salat 2004; Fjell and Walhovd 2010; Storsve 2014; Frangou 2021; Bethlehem 2022), and annual thinning is around 0.35% across the cortex (Storsve 2014), with maximum thinning estimated around 0.07 mm per decade (Salat 2004). Regionally, the frontal cortex is generally reported to see more thinning than other areas (Salat 2004; Fjell and Walhovd 2010; Thambisetty 2010; Storsve 2014). Cortical surface area has also been shown to decrease with age (Salat 2004; Storsve 2014; Bethlehem 2022), with a mean annual change of 0.19% and the biggest changes seen in the medial temporal, occipital, and posterior cingulate cortices (Storsve 2014). Overall, with the exception of the first decade, a monotonic reduction of average thickness, volume, and surface area is seen, with little regional differences in these trends.

Prior studies describing lifespan effects on cortical morphology combined information from all length scales down to the “native scale” of the brain surface reconstruction. Such analyses thus lacked a delineation between morphological changes happening at specific scales. However, it has been shown that morphological effects of aging diverge at larger scales (Wang 2023). We therefore suggest that a multiscale analysis is necessary to capture the age-related changes at the level of small cortical features (single gyrus) separately from the more extensive effects happening at larger scales (lobes and hemisphere level). We hypothesize that such age-related changes occurring at different scales follow unique and possibly divergent trajectories across the lifespan, and may also display regional specificity.

To enable such multiscale analyses, we have recently proposed a re-conceptualization of cortical morphology (Wang 2023), in which distributed shape information is quantified across multiple length scales. Rather than measuring a single value of, eg volume of the cortex, we calculate how morphological features of different sizes separately contribute to the total value. By deleting features smaller than a desired cut-off length scale, we are able to re-render any given cortex to retain only features larger than the cut-off. By varying the cut-off scale, our focus can then range from the smallest sulci and gyri to entire cortical hemispheres. This approach enriches traditional morphological quantification by explicitly quantifying the morphology of increasingly larger features. The re-rendered cortices are shown to be plausible in terms of morphological properties and agree with neuroevolutionary data (Wang 2023).

In this study, we characterize morphological changes of the healthy lifespan past the first decade in a multiscale analysis. We begin by describing whole-brain changes, followed by regional differences in individual lobes. Finally, in a proof-of-principle study, we estimate brain age from a single scale *vs.* multiple scales. This brain age model is not optimized for performance or designed to compete with existing models, but to illustrate the added value of multiscale morphometry. Taken together, we provide a comprehensive model of the lifespan, which could serve as a normative model in the future for both individuals and cohorts of patients.

## Materials and methods

### Data and preprocessing

To study lifespan effects on cortical morphology, we used T1 weighted MRI of healthy subjects from 2 large public datasets, the Nathan Kline Institute Rockland Sample (NKI) (Nooner 2012), and the Cambridge Centre for Ageing and Neuroscience (CamCAN) dataset. Both datasets were acquired on a 3T Siemens TIM Trio scanner with a 1 mm isotropic voxel size (for more details, see https://fcon_1000.projects.nitrc.org/indi/enhanced/mri_protocol.html for NKI, [Shafto 2014; Taylor 2017] for CamCAN).

**Fig. 1:**
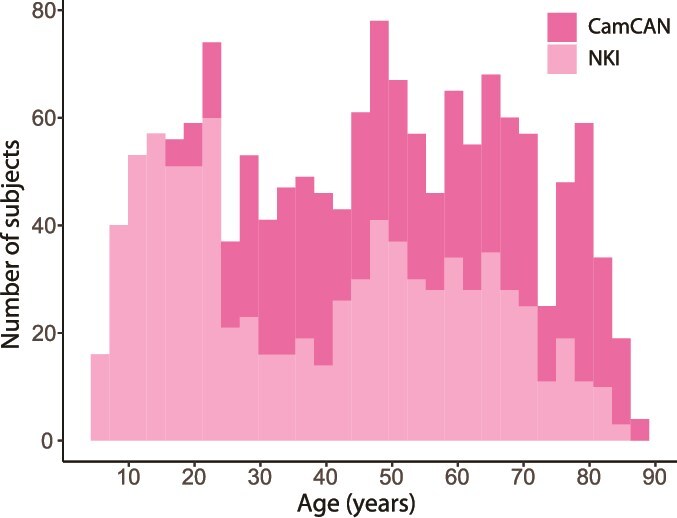
Age distribution of subjects used for this study. Total count of subjects available by age, stacked across datasets and colored by dataset.

Of the NKI dataset, 96 images were rejected due to insufficient image quality and motion artifacts, either because they failed processing, or after processing they were identified as outliers and visual inspection attributed this to the quality of the raw image. This left 833 subjects from this dataset (325/508 m/f). 641 subjects of the CamCAN dataset completed processing (327/314 m/f). The age distribution of the data used can be seen in Fig. 1.

The MRI were preprocessed with the FreeSurfer 6.0 recon-all pipeline, from which we obtained pial and white matter surface reconstructions, labeled with the Desikan-Killiany parcellation. The surfaces were visually quality checked and corrected where necessary.

All analyses were performed on anonymised data, which were acquired previously as part of other studies/consortia, with ethical approval from Newcastle University (reference: 22/SC/0016).

### Coarse-graining and regional computation of multiscale morphometrics

**Fig. 2:**
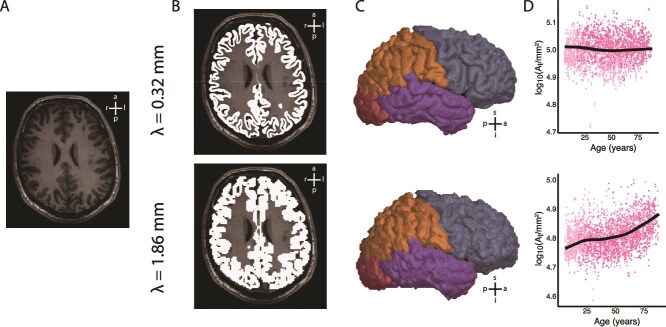
Computation of lifespan trajectories in multiscale morphometrics in cortical regions. Algorithm is shown for 2 example scales, $\lambda =$ 0.32 mm (top row) and $\lambda =$ 1.86 mm (bottom row). The algorithm was repeated for scales between 0.32 and 3 mm. (A) Axial slice of MRI from example subject. (B) Coarse-grained gray matter and white matter volumes overlayed in light gray over the MRI. (C) Reconstructed gray matter surfaces, with lobes labeled using the nearest point on the original FreeSurfer reconstruction. (D) Harmonization across sites and inference of lifespan trajectories of pial surface area $A_{t}$ with gamlss models.

We performed a coarse-graining of the cortical surfaces following the algorithm as described in Wang (2023). Briefly, the detailed pial and white matter surfaces that were reconstructed by FreeSurfer were converted to a volume, by filling them with voxels of side length $\lambda $. The voxels were labeled as being within the gray matter or the white matter (Fig. 2A). This provides a segmentation rendered at scale $\lambda $, ie gray/white matter volumes which consist of voxels of side length $\lambda $ and are therefore coarser than the original surfaces. These processing steps were performed following the pipeline in Wang (2023), for which code can be found on github (https://github.com/cnnp-lab/CorticalFoldingAnalysisTools/blob/master/Scales/). We then reconstructed surfaces over the coarse-grained volumes using MATLAB function isosurface (Fig. 2B), to obtain coarse-grained surfaces, in which folding features smaller than $\lambda $ have been removed. These surfaces are different to a downsampled rendering of the FreeSurfer surfaces, in that small folding details was intentionally removed, rather than attempting to describe the same shape using less information. We used the FreeSurfer localGI pipeline to obtain smooth (exposed) surfaces of the coarse-grained volumes with a sphere of 15 mm diameter used for the closing operation. We also obtained a convex hull over the coarse-grained pial surface.

For the hemisphere analysis, we computed the total surface area $A_{t}(\lambda )$ of the coarse-grained pial surface, the surface area of the convex hull $A_{e}(\lambda )$, and estimated the average cortical thickness $T(\lambda )$ as the ratio of the volume of the gray matter (obtained from the coarse-grained mask in Fig. 2A) and $A_{t}(\lambda )$.

We followed the FreeSurfer assignment of Desikan-Killiany regions into insula, frontal, parietal, temporal, and occipital lobes (https://surfer.nmr.mgh.harvard.edu/fswiki/CorticalParcellation). We labeled vertices on the coarse-grained pial, white matter, and exposed surfaces by the label of the closest vertex on the original pial/white matter surface (Fig. 2B). From this, we computed the lobe-wise pial surface area $A_{t}(\lambda )$ and exposed area $A_{e}(\lambda )$. We estimated the average thickness $T(\lambda )$ of each lobe as the average minimum distance from each vertex of that lobe on the pial surface to the white matter surface. The pial surface area allocated to the insula was divided into the frontal, parietal, and temporal lobe according to the relative surface area of those lobes. Likewise, we also included the insula measurement of $T(\lambda )$ into the averages of these 3 lobes according to their relative surface areas.

We repeated this algorithm for scales $\lambda $ ranging from 0.32 mm to 3.02 mm (start at $10^{-0.5}$, then each stepwise increase multiplies by $10^{0.07}$, e.g. second step is $10^{0.07}*10^{-0.5} = 0.37$). We chose this range as it covers surface reconstructions from a very close approximation of the original FreeSurfer surface ($\lambda =$ 0.32 mm), to quite a coarse surface, at which differences between individual brains are still visible ($\lambda =$ 3 mm). At the lower end of the range of scales, small changes in $\lambda $ have a larger effect on the resulting surface reconstruction, whereas at larger scales the surfaces change more gradually. Because of this, we sample nonlinearly, ie more densely from the smaller scales, to capture and describe multiscale effects thoroughly. An analysis of even larger scales can be found in Supplementary material (see Supplementary S5).

Note that the scales used for coarse-graining do not directly correspond to acquisition resolutions, for example a surface coarse-grained at $\lambda =$ 1 mm is not equivalent to the FreeSurfer surface reconstruction of a 1 mm isotropic image, but will have lost some of the folding detail. In fact, no matter how small the coarse-graining scale, it is impossible to fully reconstruct the original surface with this method, since surfaces between touching gyri walls will not be recovered.

We continued the analysis with the morphometrics average thickness $T(\lambda )$ and pial surface area $A_{t}(\lambda )$, computed at hemisphere level and for each lobe at a range of spatial scales $\lambda $.

### Analysis of lifespan effects on morphometry in cortical hemispheres and lobes

To extract the lifespan effect on different morphometrics from the 2 datasets, we used generalized additive models for location, scale, and shape (GAMLSS) from the gamlss R package (https://cran.r-project.org/web/packages/gamlss). We included the acquisition site as a random variable affecting the mean and standard deviation of the distribution, to account for scanner and scanning protocol effects and harmonize the data between sites. Since we were not primarily interested in sex differences, we accounted for these with random variables as well, modeling the effect on mean, standard deviation, and skew, but producing a single overall trajectory for both sexes (Fig. 2C). An assessment of the model fits based on sex group, as well as models trained on each sex separately can be found in Supplementary S4. The age effect was modeled on all 4 distribution parameters with smooth terms using a P-splines. Model formulas can be found in Supplementary S1. The model was fit with a mixed algorithm (“Rigby and Stasinopoulos,” then “Cole and Green”). We fit these models for hemispheres and cortical lobes, and for each scale, which allowed us to infer hemisphere and regional scale-dependent aging trajectories, whilst accounting for covariates and harmonizing between sites. We performed an analysis of the stability of models, which can be found in Supplementary S3.

### Multiscale metrics as brain age predictors

To illustrate the complementary information contained at different spatial scales, we performed a brain age estimation using only a single morphometric computed for cortical hemispheres. To correlate cortical shape to aging, one needs to extract morphometric variables from the images. The choice of the variables to use is thus, implicitly, a choice of which morphological information to retain, and which to discard.

The most general description of a cortex is that of a thin sheet of gray matter, folded in complex patterns around white matter. Total area ($A_{t}$) alone cannot tell apart, say, a smooth cortex from a tightly crumpled one; and it is known that aging affects deep and shallow sulci differently. To capture that distinction, one needs some indication of how much of this area can be attributed to folded features of different sizes. By applying the coarse-graining algorithm, we geometrically removed sulci and gyri smaller than some threshold scale, and measured the area of resulting coarse-grained cortex. Progressively increasing the threshold allowed us to quantify the contribution of the various fold sizes to the aggregate total area.

We used the mgcv R package to fit generalized additive models (GAM), with fixed effects of sex and site, and smooth terms of the predictor $A_{t}$ to estimate a subject’s age. We fit 3 models to the data: One using $A_{t}$ computed without doing any coarse-graining of the cortical surface ($\lambda = native$), a second using $A_{t}$ computed at scale 1.86 mm ($\lambda $ = 1.86 mm), and a third using $A_{t}$ computed both ways together. The specific scales were selected for the model as the “native” scale usually be used for quantitative morphometry, and a much larger scale, which previously resulted in the biggest effect size discriminating between young and older groups of subjects (Wang 2023). The 3 models were thus:


\begin{align*} \mathrm{Model \: 1: } \: Age \sim \: & s(A_{t} (\lambda = native)) + Sex + Site \nonumber \\ \mathrm{Model \: 2: } \: Age \sim \: & s(A_{t} (\lambda = 1.86\ mm)) + Sex + Site \nonumber \\ \mathrm{Model \: 3: } \: Age \sim \: & s(A_{t} (\lambda = native)) \: + \nonumber \\ & s(A_{t} (\lambda = 1.86\ mm)) + Sex + Site. \nonumber\end{align*}

Here, $s(A_{t} (\lambda ))$ is a smooth term (with a basis of thin plate regression splines) of the measure $A_{t}$ computed at scale $\lambda $, and sex and site are fixed effects. This is a very simple model, which could easily be improved by adding more metrics, scales, and regional information. We intentionally decided to keep the model simple to demonstrate the effect of adding multiscale information.

We ran 1,000 bootstraps for each model, computing the adjusted $R^{2}$ each time, to get a distribution for each model as a measure of model fit which accounts for the different degrees of freedom in the models.

## Results

### Lifespan trends measured at different scales have opposing trajectories within a single metric

**Fig. 3:**
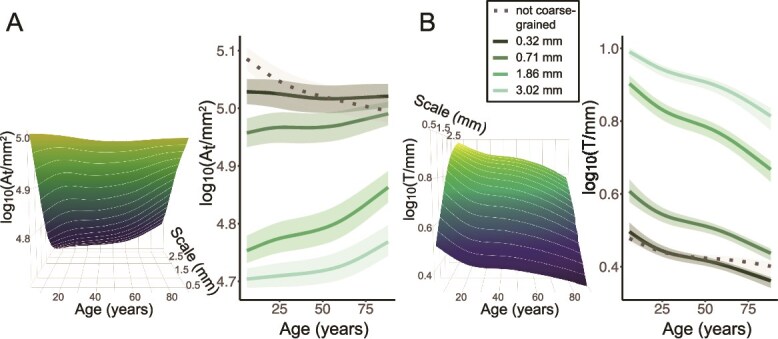
Lifespan effects on cortical hemispheres measured in multiscale morphometrics. (A) Pial surface area log10($A_{t}$/mm$^{2}$). (B) Average cortical thickness log10($T$/mm). Planes (left) show trajectories as functions of scales between 0.32 and 3 mm, where the color corresponds to the height of the plane, ie the value of $A_{t}$ or $T$. Line graphs show trajectories without coarse-graining (“native scale”, dashed line) and for 3 scales 0.32, 0.71, 1.86, and 3.02 mm, where lighter color indicates a larger scale used for the coarse-graining procedure. Shaded bands show the interquartile range of the distribution. The relative ordering of values of $T$ for the different scales results from a cortex that becomes both thicker and less gyrified as the cut-off scale increases.

The GAMLSS modeling produced lifespan trajectories in each scale and morphometric. In Fig. 3, we show trajectories for all scales as 3D “planes,” and cross-sectional trajectories for 3 representative scales (0.32, 0.71, and 1.86 mm) as line graphs. For reference, we also depict the trajectory obtained from the original, not coarse-grained (“native scale”) FreeSurfer surfaces as a dashed line. The 3 scales shown cover the range from a close approximation of the full, detailed FreeSurfer pial surface (0.32 mm), to a coarse-grained surface with few folding details remaining (1.86 mm). Cross-sectional trajectories of a wider range of scales can be found in Supplementary S5, which provide a thorough multiscale description of the general morphological changes in hemispheres across an age range of 6 to 88 years.

As expected as a result of our multiscale method, we observed an overall increase of thickness and decrease of surface area with increasing scale (ie with increasing merging of gyri) in terms of the offset for each trajectory over the lifespan (Fig. 3). However, the relative change over the lifespan within each trajectory was surprising:

Whilst the pial surface area measured at a scale of 0.32 mm remains relatively constant throughout the lifespan (<5% change), we see a surprising increase in surface area with age in the larger scales, particularly after 50+ years (Fig. 3A). For example, at 1.86 mm, we observe a 25% increase in surface area across the sampled lifespan. These trajectories are in contrast to that observed in the “native scale,” which is monotonically decreasing throughout the lifespan as expected and widely reported.

We further observed a steep decrease of average cortical thickness throughout childhood, and a slower decrease in adulthood visible in all scales (Fig. 3B). We note, however, that the rate of decrease is most pronounced in larger scales, eg at 1.86 mm, we observe a nearly 60% decrease compared to the 40% at 0.32 mm across the sampled life span.

In addition to commonly used metrics of cortical thickness and surface area, we also explored lifespan effects in a set of new morphology measures that are statistically independent (Wang 2021). Supplementary S2 lays out the rationale for considering independent metrics, their computation, and hemisphere and regional lifespan effects.

### Regional differences in lifespan effects become apparent in larger scales

**Fig. 4:**
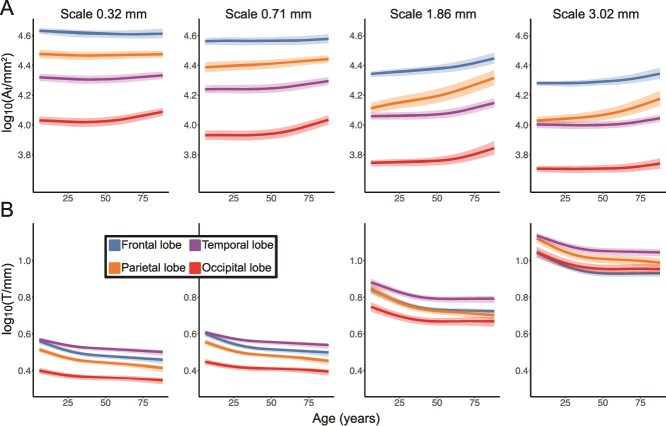
Lifespan effects in main lobes measured in multiscale metrics. Columns show trajectories in spatial scales 0.32, 0.71, 1.86, and 3.02 mm. (A) Pial surface area log10($A_{t}$/mm$^{2})$. (B) Average cortical thickness log10($T$/mm). Colors indicate individual trajectories of cortical lobes. Shaded bands show the interquartile range of the distribution.

Next, we investigated lobe-wise lifespan effects across scales. We found that lobe-level changes of pial surface area $A_{t}$ across the life span were mostly reflected in the coarse scale of 1.86 mm, where most small folding details have been removed. Here, we saw a sharper incline in the parietal lobe across the lifespan (approx. 60%), and a shallower trajectory in the temporal lobe (approx. 20%, see Fig. 4A).

In cortical thickness $T$, we found slight regional differences in trajectory in childhood: eg at scale 0.32 mm, the cortex thins quicker in the frontal and parietal lobes compare to temporal and occipital lobes. However, lobal differences during adulthood were minimal, and we gained little additional information across different scales (Fig. 4B).

### Brain age estimate from $A_{t}$ is improved by using morphometrics from multiple scales

As a proof-of-principle, to demonstrate the added value of multiscale morphometrics in an application, we carried out a simple brain age estimation using only the metric of pial surface area $A_{t}$. Note this brain age model is not optimized for performance or designed to compete with existing models, but only to illustrate the added value of multiscale morphometry.

**Fig. 5:**
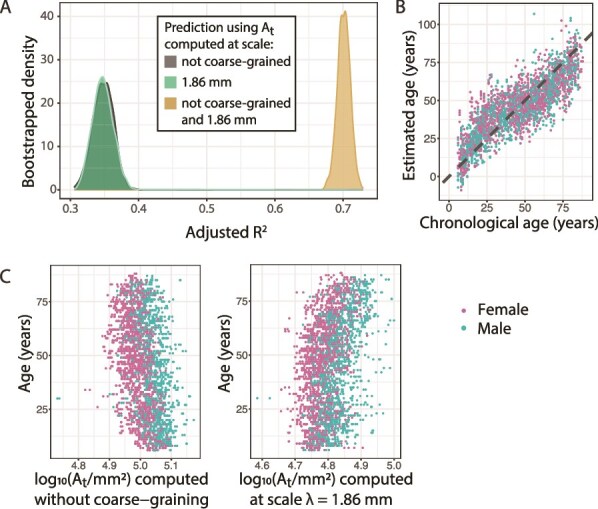
Brain age estimation using surface area, sex, and site. (A) Distributions of bootstrapped adjusted $R^{2}$ obtained from GAM brain age model incorporating the effects of age, site, and pial surface area computed either without coarse-graining (“native” scale), at scale 1.86 mm, or using both scales. (B) Fit of model using both $A_{t}$ computed without coarse-graining and $A_{t}$ computed at scale 1.86 mm, showing predicted brain age versus actual (chronological) age. (C) Data underlying the brain age models, showing subject age versus pial surface area ($A_{t}$) computed without coarse-graining (left) and computed at scale $\lambda = 1.86$ mm (right).

We compared model performance when using a single morphometric to using multiple scales (Fig. 5). Both of the models using $A_{t}$ computed at a single scale (“native”, ie not coarse-grained and 1.86 mm, respectively) had a low adjusted $R^{2}$ of around 0.35. However, using surface area measurements computed both without coarse-graining and at scale 1.86 mm, the model fit was improved to an adjusted $R^{2}$ of around 0.7, showing that model performance was enhanced by using just a single metric at different scales. The reason becomes apparent when looking at the data underlying the models (Fig. 5C): $A_{t}$ computed at both scales correlates with age, but with opposite signs, therefore using both adds complementary information to the model prediction.

As a side note, a straight line fit through the data in Fig. 5B would have a slope lower than 1, indicating that our model displays a slight bias to the mean age of the dataset (48 years). This “regression to the mean” is a known phenomenon in brain age estimation, that can be accounted for by performing an age regression on the estimated values before further analysis (Le 2018; Liang et al. 2019). Since we are not using the estimated brain age for further applications here, we omitted this step.

## Discussion

In this study, we analyzed lifespan effects on cortical morphology. We quantified changes in a range of morphological metrics across spatial scales, and found divergent trajectories with increasing age at different scales. Furthermore, we also observed unique trajectories in different lobes at larger spatial scales. Our findings enrich current reports in the literature, which have focused only on a single “native” scale analysis, and mostly found monotonically decreasing lifespan trajectories. Clearly, different types of morphology information are contained at different spatial scales, as evidenced by the divergent lifespan trajectories. As a proof-of-principle, we also demonstrated that these different types of information can be leveraged to improve predictive performance.

### Multiscale cortical morphometry

Here, we used a multiscale approach based on computationally coarse-graining the surface representation of the brain. This allows us to create coarse-grained biologically plausible (Wang 2023) reconstructions of the brain for specific length scales, from which we can compute scale-specific morphometrics. Like any surface representation of the brain, the re-rendered surfaces are an abstraction of the real surface of the cortical tissue. We always apply some scale through which we reconstruct it, but rather than using only the smallest scale we can achieve, multiscale morphometry allows us to also analyze larger-scale representations of the brain.

Scale-specific morphometrics allow us to disentangle the shape information of features of varying size, since specific processes and pathologies might affect cortical shape at different scales. For example, lesions may show as localized alterations at small scales, whilst dementias might cause atrophy in wide areas and large-length scales. In future applications, this new quantification of morphology could improve methods for diagnosis, whilst also being more precise in delineating diverging morphological trajectories.

Other complementary multiscale descriptions of cortical morphology have also been proposed, such as spectral analysis of cortical geometry (Chen 2022). This approach also characterizes cortical morphology across spatial scales, but assumes basis functions, and the interpretation of eigenvalue spectra is thus challenging due to possible harmonics caused by the specific choice of the basis function. Additionally, key differences to our approach include: intrinsic co-variates (such as isometric brain size) are not factored out in a way that respects scaling relationships, and the cortical shape is only analyzed for the white matter surface. Nevertheless, we are excited that other researchers have also turned their attention to multiscale morphometrics. We believe that future iterations of this approach will combine multiple methods and open the door to exciting discoveries in morphological analysis of (human) brains.

### Lifespan trends in cortical hemispheres

At the smallest scales, our surface reconstruction is a close approximation to the detailed FreeSurfer surface, and indeed the lifespan trajectories we observed at a scale of 0.32 mm agree with previous descriptions of lifespan effects (Frangou 2021; Bethlehem 2022) and our trajectories from the original not coarse-grained surfaces. In the hemisphere analysis, the coarse-graining affects average cortical thickness mostly by changing the offset rather than the shape of the trajectory. This indicates that lifespan effects in this metric are largely scale-invariant, meaning cortical thinning affect all scales similarly.

However, in pial surface area at different scales, we found unique, diverging lifespan trajectories. For example, whilst the not coarse-grained lifespan trajectory is monotonically decreasing, the trajectory measured at scale $1.86$ mm monotonically increases with age. This shows that this metric is able to capture changes in cortical shape uniquely in different scales. It can be interpreted as surface area loss from the detailed gyri and sulci, and surface area gain in the larger (multiple gyri or lobal level) structures. This observation agrees with the reported sulci widening with advancing age (Liu 2010), and this effect appears to be best captured in larger spatial scales. Such scale-dependent lifespan effects cannot be derived or observed from traditional morphometry approaches operating at a single scale and represent a key contribution of our work here.

### Regional differences in age trends

Our multiscale analysis of age trends in cortical lobes revealed some regional differences that were not detectable at the smallest scales alone. We found that pial surface area increased with age in the occipital lobe even in the smallest scales, likely caused by the tight cortical folds here, which accelerate the coarse-graining procedure. We found minimal regional differences in average cortical thickness, but what we did find is in agreement with existing literature. Namely, a difference in offset of trajectories, and steeper and longer slope during development in frontal and parietal cortices (Frangou 2021).

Overall, we found that additional scales contain important, new, nonredundant information, making them able to distinguish regional lifespan effects much better than traditional metrics and computation methods. Further, they are possibly pointing to biological processes in lifespan affecting the shape of cortical regions differently.

### Utilizing multiscale information

We built a model for brain age estimation using only sex, scanning site, and pial surface area as predictors. The estimates were vastly improved by including surface area measurements from 2 scales, rather than just a single measurement at one scale. The model’s performance does not approach that of current best brain age estimators using morphometrics, which achieve mean absolute errors of under 4 years, for example by combining information from multiple modalities (Cole 2020; Rokicki et al. 2021; Guan et al. 2023). Whilst our model could quickly be improved by adding more metrics, scales, and regions, we kept it simple, to highlight the complementary information that is contained at different spatial scales and the value we can extract from it. Our aim was not to optimize prediction performance or compete with existing models, but to demonstrate that even within a single morphometric, an analysis of multiple scales offers unique information relevant to age. We acknowledge that a well-optimized single-scale model, especially one leveraging multiple metrics and brain regions, will likely outperform our multiscale approach in terms of accuracy. However, the key insight here is not that multiscale information is superior overall, but that it provides additional explanatory value beyond any single scale. It suggests potential for future brain age estimators, which build on this result, creating models that are both accurate and interpretable. Of course, brain age estimation is just an illustrative application area of multiscale morphometry, and our results suggest that analyses in other applications, such as diagnosis or structural abnormality detection, might be improved with a multiscale analysis approach.

### Normative models of healthy aging

Normative models are the mapping of health-related variables to each other, and result in population-level trajectories (Rutherford 2023). Previously, normative models have been built for various brain morphology metrics (Bethlehem 2022; Ge 2024; Little 2025). Whilst they capture variation in the population, they also allow us to quantify individual variation and deviation from the population. This both enhances group-level inference, but also enables subject-level analysis, for example in MRI analysis finding atypical trajectories of structural change (Rutherford 2022). Hence, they can help contrast trajectories of degenerative diseases, show how and when they diverge from healthy aging, and describe changes in disease cohorts, isolated from aging.

In our study, we built multiscale models of morphometrics computed at a range of spatial scales. This multiscale approach has the potential to improve sensitivity and specificity of the model, since previous normative models based on multimodal data have shown improved performance due to the complementary information contained in the data (Kumar et al. 2023).

### Limitations

Even though our inference of lifespan effects is based on 2 large data sets, the analysis could be improved by including data from additional sites, making the trajectories more robust and generalizable. In particular, the effects in ages below 18 years were only based on the NKI data. In future work, we will add more developing cohorts so that the analysis is not based on a single dataset for this age range.

Currently, our regional analysis is limited to cortical lobes. We were able to show local lifespan effects in larger scales at this level already, but we will develop methods to further localize the computation of multiscale morphometrics for more precise regional analyses in the future.

### Conclusion

Our study describes healthy lifespan effects on multiscale morphometrics. We found that metrics computed at different spatial scales capture distinct aspects of cortical shape, suggesting that there could be different biological mechanisms underlying the effects that uniquely impact individual scales and cortical regions. We also show that larger spatial scales should be leveraged for their additional information in morphometric analyses.

## Supplementary Material

SupplementaryMaterials_bhaf154

## Data Availability

The analysis was carried out on public datasets, see http://rocklandsample.org/ for NKI and https://www.cam-can.org/ for CamCAN. The coarse-graining of cortical surfaces was performed using code available on github: https://github.com/cnnp-lab/CorticalFoldingAnalysisTools/blob/master/Scales/.
