# Supplementary material for: Multiscale cortical morphometry reveals pronounced regional and scale-dependent variations across the lifespan
Source: Cereb Cortex. 2025 Jun 23;35(6):bhaf154. doi: 10.1093/cercor/bhaf154 (PMC12205997; doi:10.1093/cercor/bhaf154)
Supplement: SupplementaryMaterials_bhaf154 [file supplementarymaterials_bhaf154.pdf]

Supplementary Materials

Multiscale cortical morphometry reveals pronounced regional and scale-dependent variations across the lifespan

Karoline Leiberg[1], Timo Blattner[2], Bethany Little[1], Victor B.B. Mello[2], Fernanda H.P. de Moraes[3,4], Christian Rummel[2], Peter N. Taylor[1,5,6], Bruno Mota[3], and Yujiang Wang[1,5,6*]

1. CNNP Lab (www.cnnp-lab.com), Interdisciplinary Computing and Complex BioSystems Group, School of Computing, Newcastle University, Newcastle upon Tyne, United Kingdom
2. Support Center for Advanced Neuroimaging (SCAN), University Institute of Diagnostic and Interventional Neuroradiology, University of Bern, Inselspital, Bern University Hospital, Bern, Switzerland
3. metaBIO Lab, Instituto de Física, Universidade Federal do Rio de Janeiro (UFRJ), Rio de Janeiro, Brazil
4. Brain Connectivity Unit, D'Or Institute of Research and Education (IDOR), Rio de Janeiro, Brazil
5. Faculty of Medical Sciences, Newcastle University, Newcastle upon Tyne, United Kingdom
6. UCL Queen Square Institute of Neurology, Queen Square, London, United Kingdom

* Yujiang.Wang@newcastle.ac.uk

S1 Model formula

We modelled the morphological data using flexible sinh-arcsinh (shash) distributions. This distribution accommodates non-normal data modelling, allowing the first four moments to vary as functions of explanatory variables. All metrics were log-transformed prior to statistical analysis. Using gamlss, we modelled the distribution's parameters (moments) as dependent on explanatory variables, specifically sex, age, and scanning site. We fitted this model to each metric separately. The model formulae we used were:

$$
\begin{aligned}
&\mu \sim 1 + (1|sex) + s(age) + (1|site) \\
&\sigma \sim 1 + (1|sex) + s(age) + (1|site) \\
&\nu \sim 1 + (1|sex) + s(age) \\
&\tau \sim 1 + s(age)
\end{aligned}
$$

- The mean (μ) depends on sex (random effect), site (random effect), and a smooth function of age.
- The standard deviation (σ) depends on sex (random effect), site (random effect), and a smooth function of age.
- The skew (ν) depends on sex (random effect) and a smooth function of age.
- The kurtosis (τ) depends on a smooth function of age.

S2 Independent morphometrics

Beyond the commonly used metrics of thickness and surface area, we also applied a set of independent morphometrics described previously (Wang et al., 2021). Briefly, a scaling law of cortical folding has been proposed (Mota and Herculano-Houzel, 2015) and empirically validated (Wang et al., 2016, 2019; Leiberg et al., 2021). The scaling law allows the definition of a set of new morphometrics, that are independent of each other and have physically meaningful interpretations

(Wang et al., 2021). Healthy lifespan trajectories have also been inferred in these new morphometrics (de Moraes et al., 2022). We performed morphological analysis in these new metrics alongside the commonly-used metrics of thickness and surface area in our analyses.

S2.1 Computation

Specifically, we computed two dimensionless, independent morphometrics $K(\lambda)$ and $S(\lambda)$ as linear combinations of the logarithms of $T(\lambda)$, $A_t(\lambda)$, and $A_e(\lambda)$. We interpret these terms as the summary expressions of, respectively, a near-invariant tension term associated with the conserved dynamics of axonal elongation in a white matter surrounded by a self-avoiding grey matter; and a varying shape term that summarizes the morphological complexity of a gyrified structure. These metrics account for the covariance in A_t, A_e, and T, and we have found them to be useful for describing cortical shape, being better at differentiating morphological processes and capturing more subtle changes in cortical shape (Wang et al., 2021; Leiberg et al., 2021).

These metrics were initially well-defined for complete cortical hemispheres. To derive these metrics for individual cortical lobes, we computed the integrated Gaussian curvature over the convex hull for each lobe, and used it to correct the surface area measures to what their value would be for an entire cortical hemisphere, as per the method and reasoning laid out in (Wang et al., 2019; Leiberg et al., 2021).

After the Gaussian curvature correction of surface areas $A_t(\lambda)$ and $A_e(\lambda)$, we computed $K(\lambda)$ and $S(\lambda)$ as linear combinations of the logarithms of $A_t(\lambda)$, $A_e(\lambda)$, and $T(\lambda)$:

$$K(\lambda) = \log A_t(\lambda) + \frac{1}{4}\log T(\lambda)^2 - \frac{5}{4}\log A_e(\lambda), \tag{1}$$

$$S(\lambda) = \frac{3}{2}\log A_t(\lambda) - \frac{9}{4}\log T(\lambda)^2 + \frac{3}{4}\log A_e(\lambda). \tag{2}$$

We normalised values of K and S by the lengths of the vectors $\vec{\kappa} = \{1, \frac{1}{4}, -\frac{5}{4}\}$ and $\vec{\sigma} = \{\frac{3}{2}, -\frac{9}{4}, \frac{3}{4}\}$. That way, the transformation from traditional measures to independent measures K and S corresponds to a change in morphological coordinate space to an orthonormal basis of $\frac{\vec{\kappa}}{|\vec{\kappa}|}$, $\frac{\vec{\sigma}}{|\vec{\sigma}|}$, and $\frac{\vec{\iota}}{|\vec{\iota}|}$, where $\vec{\iota} = \{1, 1, 1\}$ is the cross product of $\vec{\kappa}$ and $\vec{\sigma}$.

S2.2 Lifespan effects on hemisphere

Figure S2.1 describes lifespan effects on two independent morphometrics: the tension term K measured in smaller scales decreases rapidly until early adulthood (about 35 years), when it plateaus and then decreases further in older age (after 70 years) (Fig. S2.1 A). In larger scales, we found an opposing trajectory that decreased only slightly until adulthood, and then increased after 60 years until older age. In the shape term S, we found similar trajectories in all scales, showing a steeper increase in adolescence, which flattened in adulthood, before becoming steeper in larger scales in later life (Fig. S2.1 B).

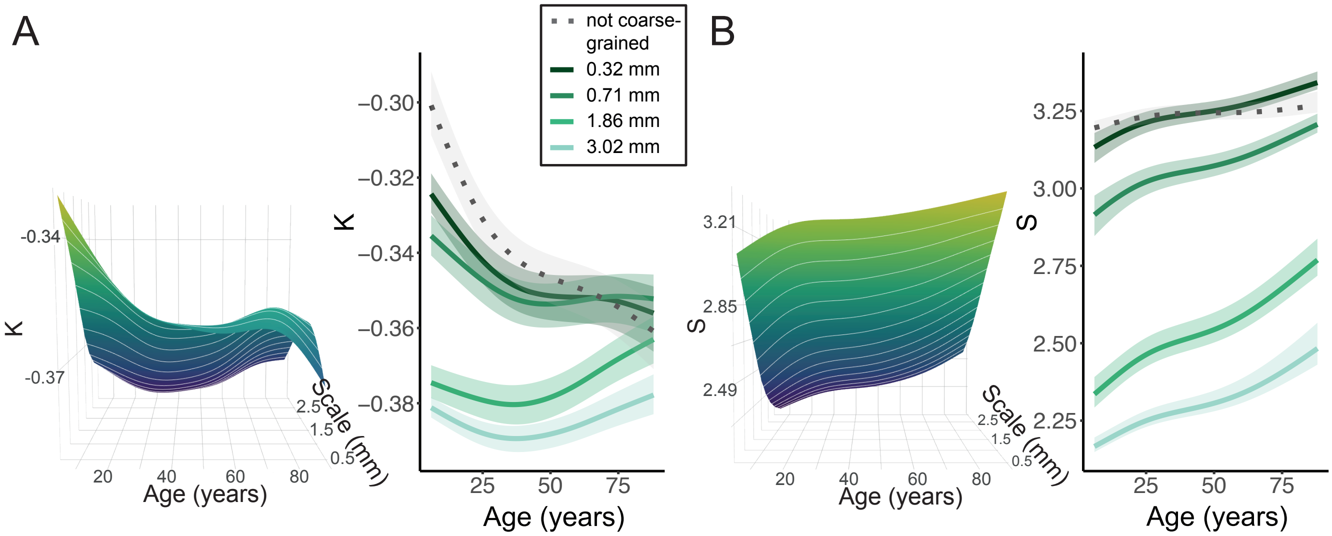

Figure S2.1: Lifespan effects on cortical hemispheres measured in multiscale morphometrics. **A)** Dimensionless metric K. **B)** Dimensionless metric S. Planes (left) show trajectories as functions of scales between 0.32 mm and 3 mm. Line graphs show trajectories without coarse-graining ("native scale", dashed line) and for three scales 0.32 mm, 0.71 mm, 1.86 mm, and 3.02 mm. where lighter colour indicates a larger scale used for the coarse-graining procedure. Shaded bands show the interquartile range of the distribution. The relative ordering of values of S for the different scales results from a cortex that becomes both thicker and less gyrified as the cut-off scale increases.

S2.3 Lifespan effects on lobes

In tension term K, regional differences in lifespan effects already become apparent at the smaller scale of 0.32 mm, where most folding details of the original surface are retained, differentiating frontal and parietal from occipital and temporal regions (Fig. S2.2 A). In the medium scale (0.71 mm), at which smaller folds have closed, but the surface still has much gyrification, the frontal

and parietal lobes saw little overall change over the lifespan, whilst temporal and occipital lobes see an increase in this morphometric and scale in later age, similar to the hemisphere trajectory in the largest scale (1.86 mm, Fig. S2.1). In S (Fig. S2.2 B), measuring morphological complexity, we again found diverging trajectories. For example, whilst we see similar lifespan effects in the occipital lobe and temporal lobe at 0.32 mm and 0.71 mm, they have unique trajectories at 1.86 mm, demonstrating again how regional differences in lifespan become more apparent at larger scales that capture overarching morphological features.

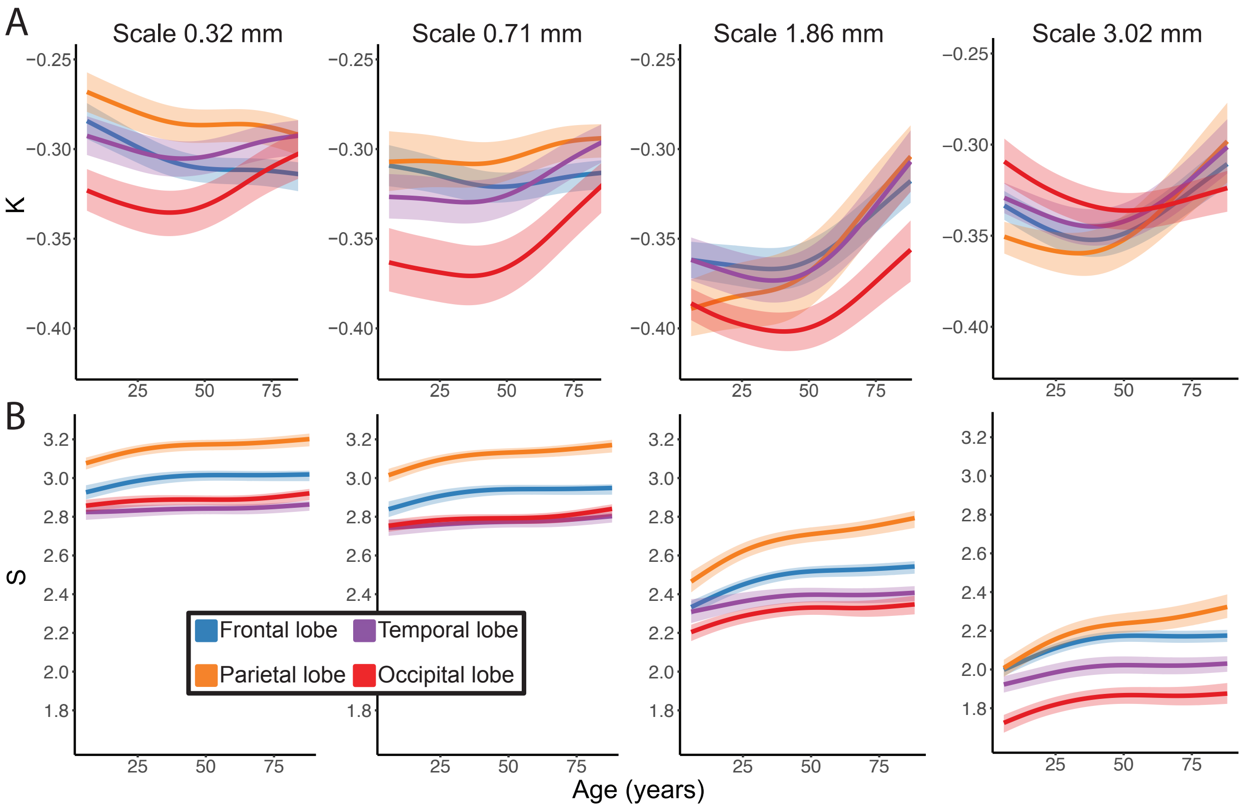

Figure S2.2: Lifespan effects in main lobes measured in multiscale metrics. Columns show trajectories in spatial scales 0.32 mm, 0.71 mm, 1.86 mm, and 3.02 mm. **A)** Dimensionless metric K. **B)** Dimensionless metric S. Colours indicate individual trajectories of cortical lobes. Shaded bands show the interquartile range of the distribution.

S2.4 Interpretation

In independent metrics K and S, the findings at the smallest scale agreed with previously described lifespan effects without any coarse-graining (de Moraes et al., 2022). We found that increased scales mainly affected the trajectory of the cortical surface tension, but only the offset of a measure of

morphological complexity. The tension term K reduces with age at small scales, but not in larger ones. This could have a biological explanation, where certain types of axons, responsible for smaller morphology features, degrade more with age. Previous literature has shown a differential effect of age in long-range fibres compared to superficial white matter (Schilling et al., 2023b,a). However, these associations are purely speculative at this stage, and future work will have to interrogate these directly.

Regional differences in age trajectories are already apparent at small scales in the independent metrics K and S. Generally, the trajectories of the frontal lobe were similar to those of the parietal lobe, and the occipital was similar to the temporal lobe, indicating differing lifespan effects between sensory and association cortices. K remains largely unchanged at scale 0.71 mm in frontal and parietal lobes, but increases with age in occipital and temporal lobes, which may result from axonal tension in medium-length white matter tracts varying more across the lifespan in the latter two lobes. This would be contrary to some previous work on regional differences in ageing-related white matter changes, which indicated reduced integrity of prefrontal white matter (Gunning-Dixon et al., 2009). However, longitudinal studies did not find such regional differences (Barrick et al., 2010). The regional differences in S show that, even though thinning is similar across regions, folding complexity changes more in the frontal and parietal regions.

S3 Model stability

Previous normative models of cortical morphometry have suggested that sample sizes of over 3000 achieve stable performance (Ge et al., 2024). Our sample of 2948 observations across both hemispheres is close, but we additionally evaluated the stability of our models. We examined correlations of fitted values on a held-out test set obtained from models trained on subsets of the full dataset and the full dataset. We also assessed error convergence of these subsampled models on the held-out test data. Figure S3 shows these tests for models of hemisphere pial surface area (relating to the trajectories in Figure 3 A), Figure S3 shows tests for models of hemisphere average cortical thickness (relating to the trajectories in Figure 3 B). We found that for both metrics, models trained on subsets of at least 1500 observations maintain a high correlation (over 99%)

with the full dataset model in terms of test set residuals (left column in each figure), indicating that predictions from the subset-trained models closely align with those from the full model. We also found that the mean absolute error of test set predictions stabilizes early (right column in each figure), suggesting that larger sample sizes to not significantly improve performance. These results confirm our models trained on the full data are stable.

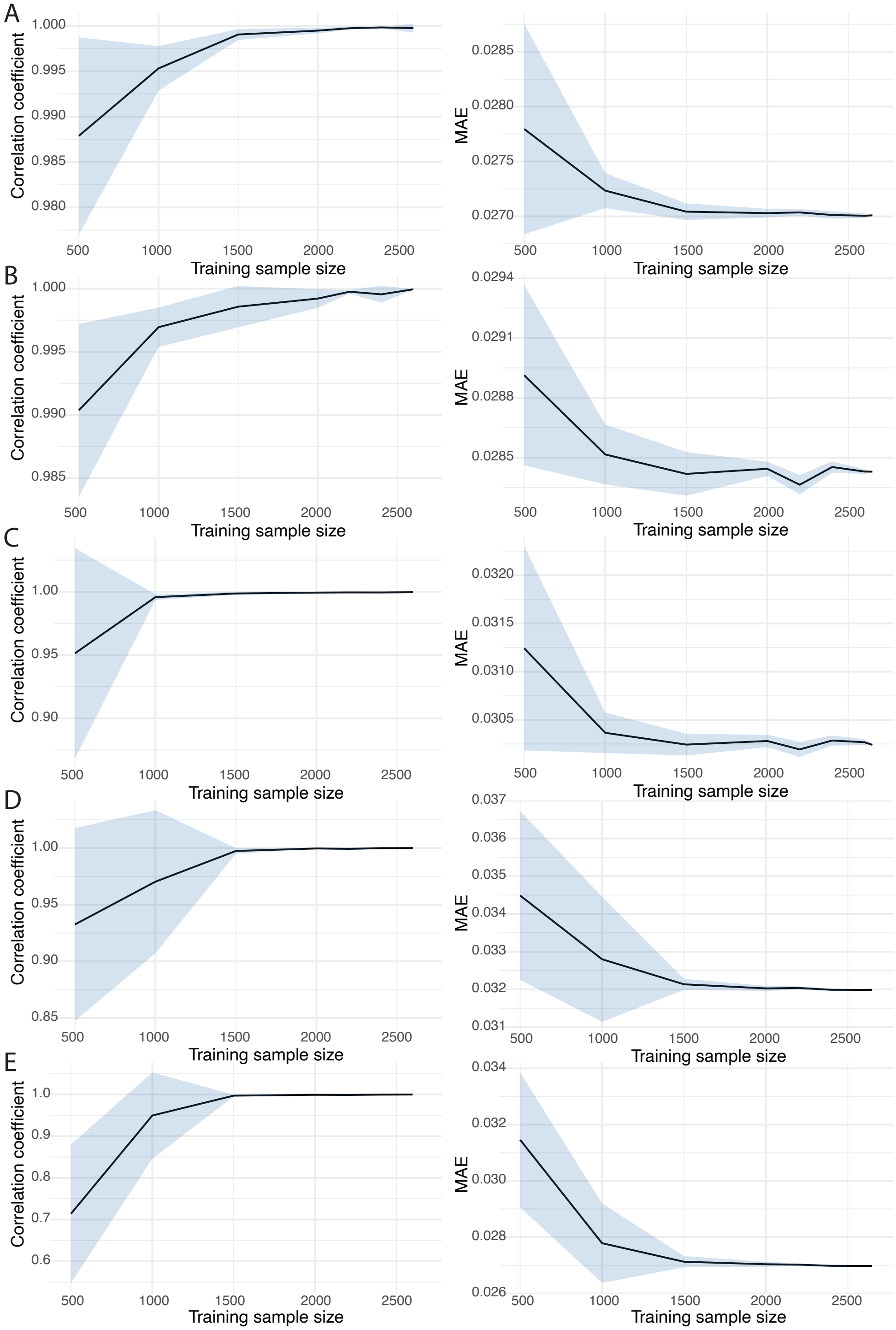

Figure S3.1: Stability of models for hemisphere pial surface area. Models of log-transformed hemisphere pial surface area were retrained on subsamples of the full dataset, with 10 repeated samples at each size. To evaluate model consistency and fit, 300 observations were randomly held out throughout. Left column: Pearson correlation between the residuals of the held-out observations from the subsampled models and the full model (n=2948), with shading representing the 95% confidence interval. Right column: Mean absolute error (MAE) for predictions on the 300 held-out observations, with grey shading indicating the 95% confidence interval. Panels **A** to **E** relate to models fit without coarse-graining, and for scales 0.32 mm, 0.71 mm, 1.86 mm, and 3.02 mm respectively.

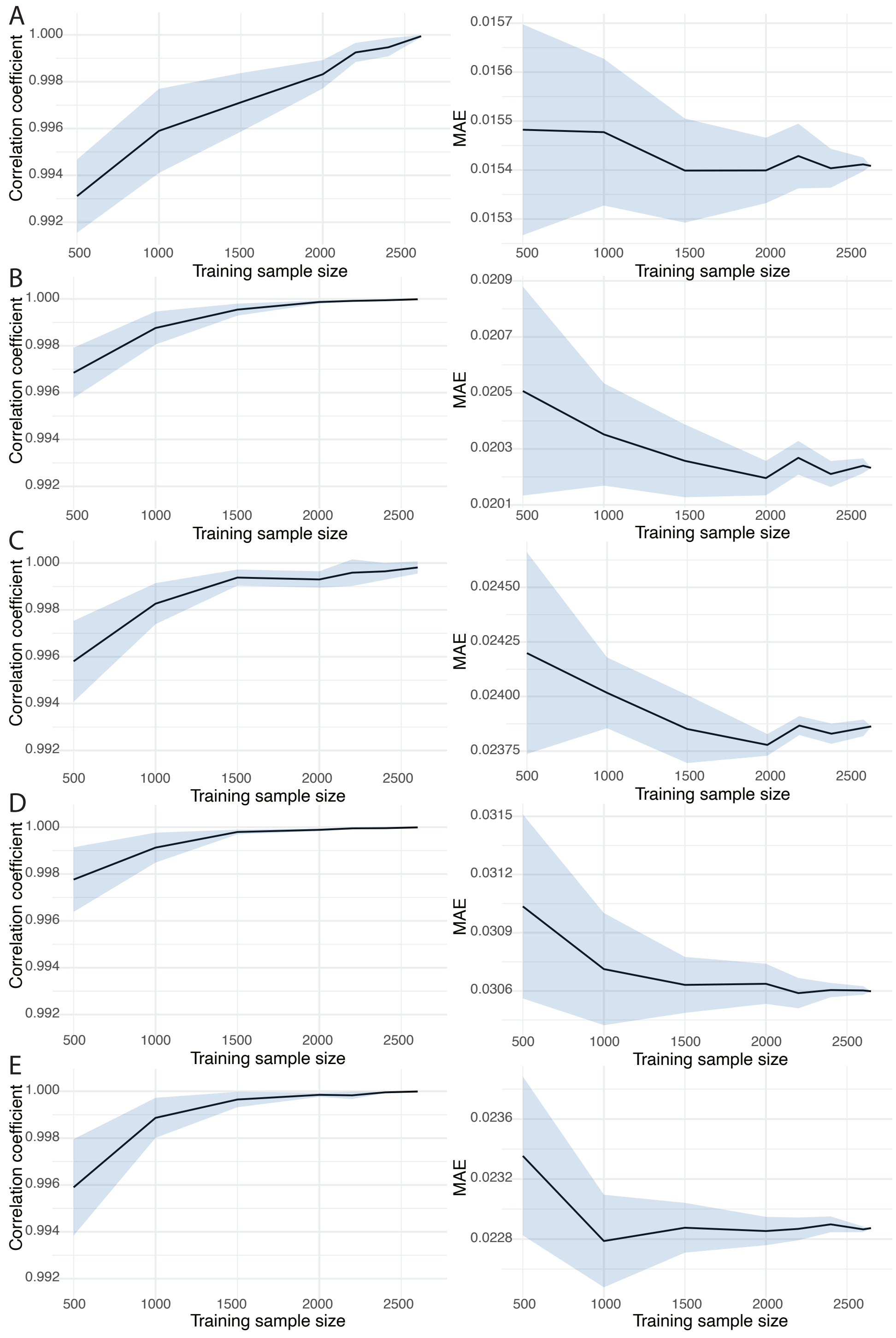

Figure S3.2: Stability of models for hemisphere average thickness. Models of log-transformed hemisphere average cortical thickness were retrained on subsamples of the full dataset, with 10 repeated samples at each size. To evaluate model consistency and fit, 300 observations were randomly held out throughout. Left column: Pearson correlation between the residuals of the held-out observations from the subsampled models and the full model (n=2948), with shading representing the 95% confidence interval. Right column: Mean absolute error (MAE) for predictions on the 300 held-out observations, with grey shading indicating the 95% confidence interval. Panels **A** to **E** relate to models fit without coarse-graining, and for scales 0.32 mm, 0.71 mm, 1.86 mm, and 3.02 mm respectively.

S4 Model effects of sex

In our models, we accounted for sex effects as an offset modelled on the parameters mean, standard deviation, and skew. Age and site effects were therefore shared between sex groups. To assess the fit of the models sex-specifically, we investigated model residuals for the hemisphere models of pial surface area and average cortical thickness (Fig. S4). We found no systematic differences in these residuals between sex groups, indicating that the models fit similarly well for male and female subjects.

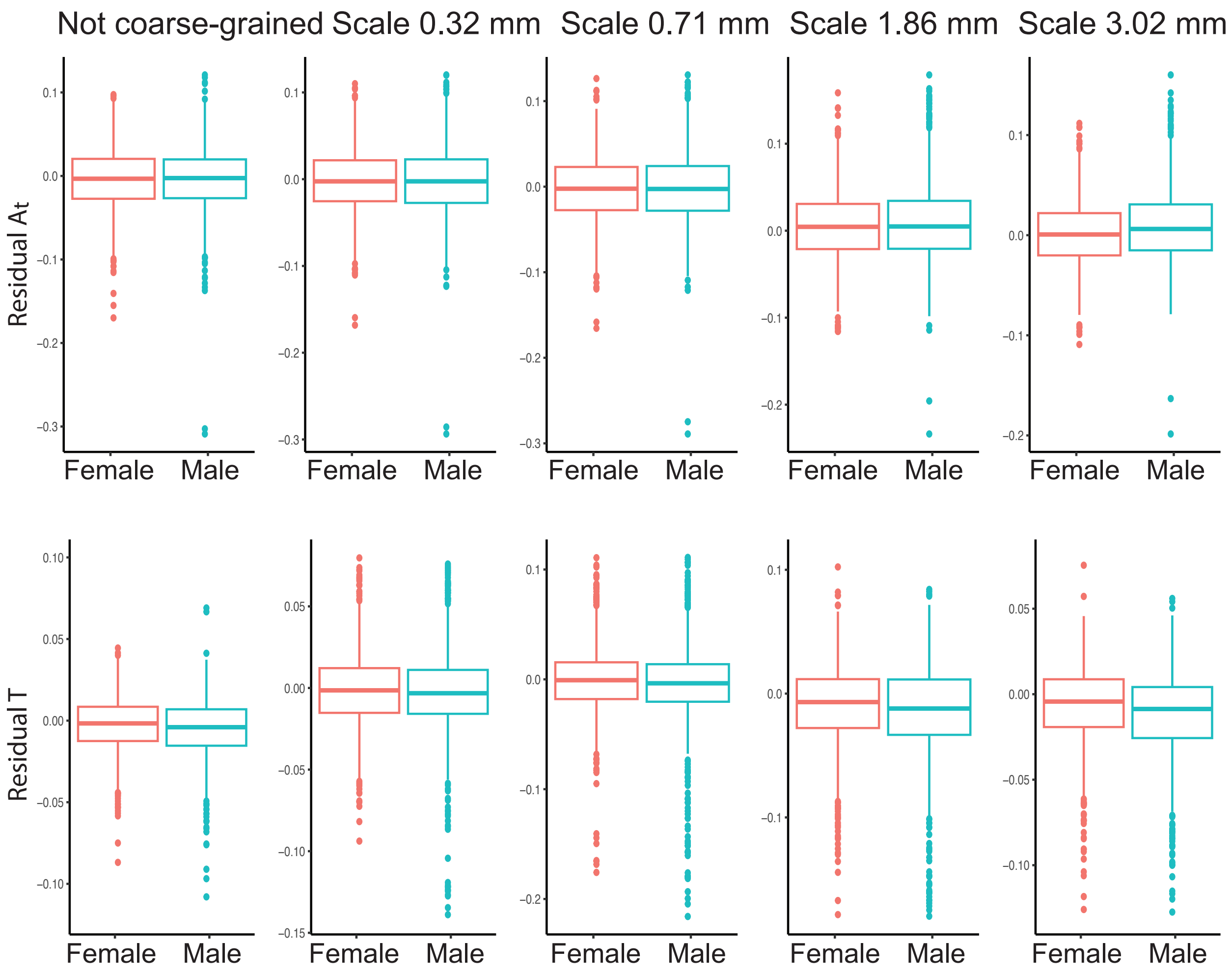

Figure S4.1: Sex-specific residuals of hemisphere models. Model residuals for each model of the hemisphere analysis are shown as box plots, grouped and coloured by sex. Top row shows models for pial surface area, bottom row shows models for average cortical thickness. Columns correspond to models for metrics computed without coarse-graining, and for scales 0.32 mm, 0.71 mm, 1.86 mm, and 3.02 mm.

Additionally, we trained models on male and female subjects separately (Fig. S4). We kept the model formula as above, but removed sex effects. Even though the models were trained on

roughly half of the full datasets and did not all achieve convergence, we found that the results of the sex-specific analysis replicated the findings based on the full datasets. Namely, the difference in trajectories between scales is retained in female/male-only models.

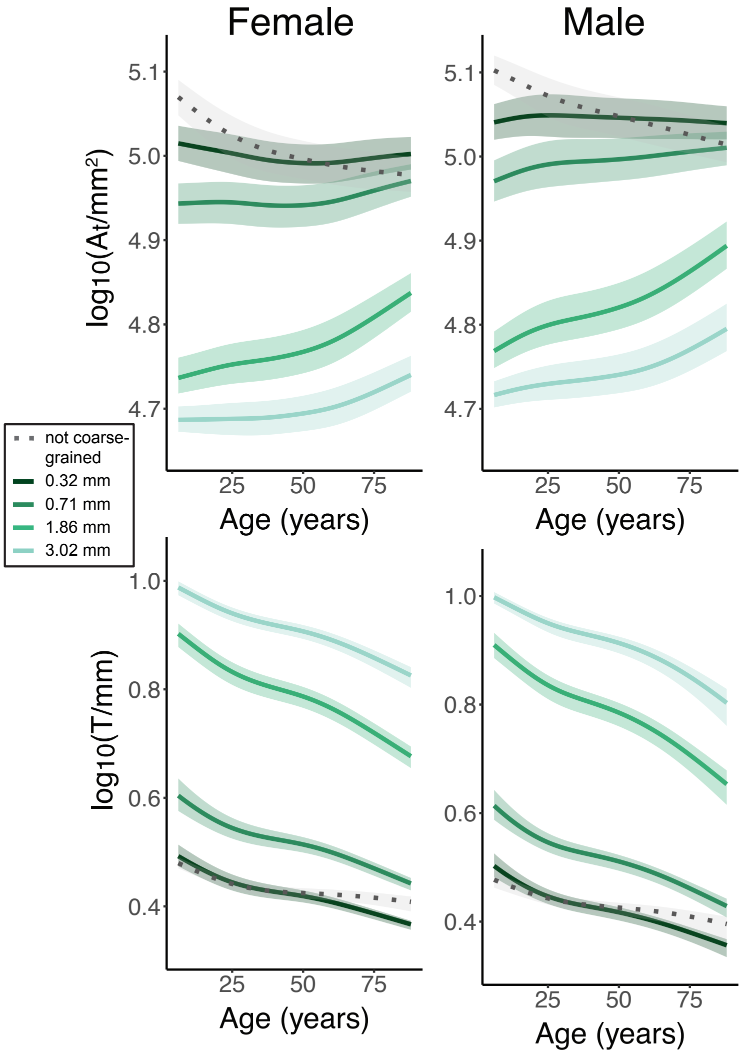

Figure S4.2: Sex-specific residuals of hemisphere models. Model residuals for each model of the hemisphere analysis are shown as box plots, grouped and coloured by sex. Top row shows models for pial surface area, bottom row shows models for average cortical thickness. Columns correspond to models for metrics computed without coarse-graining, and for scales 0.32 mm, 0.71 mm, 1.86 mm, and 3.02 mm.

S5 Lifespan trajectories in larger range of scales

For cortical hemispheres, we show "planes" of ageing effects measured across spatial scales in metrics cortical thickness, pial surface area, tension K, and shape S (Figure S5). Note that above a scale of around 5 to 6 mm, the trajectories do not differ much between scales, indiciating that

at those large scales the coarse-graining algorithm has smoothed the cortical surfaces, and further incease in scale does not alter the resulting surface reconstruction much.

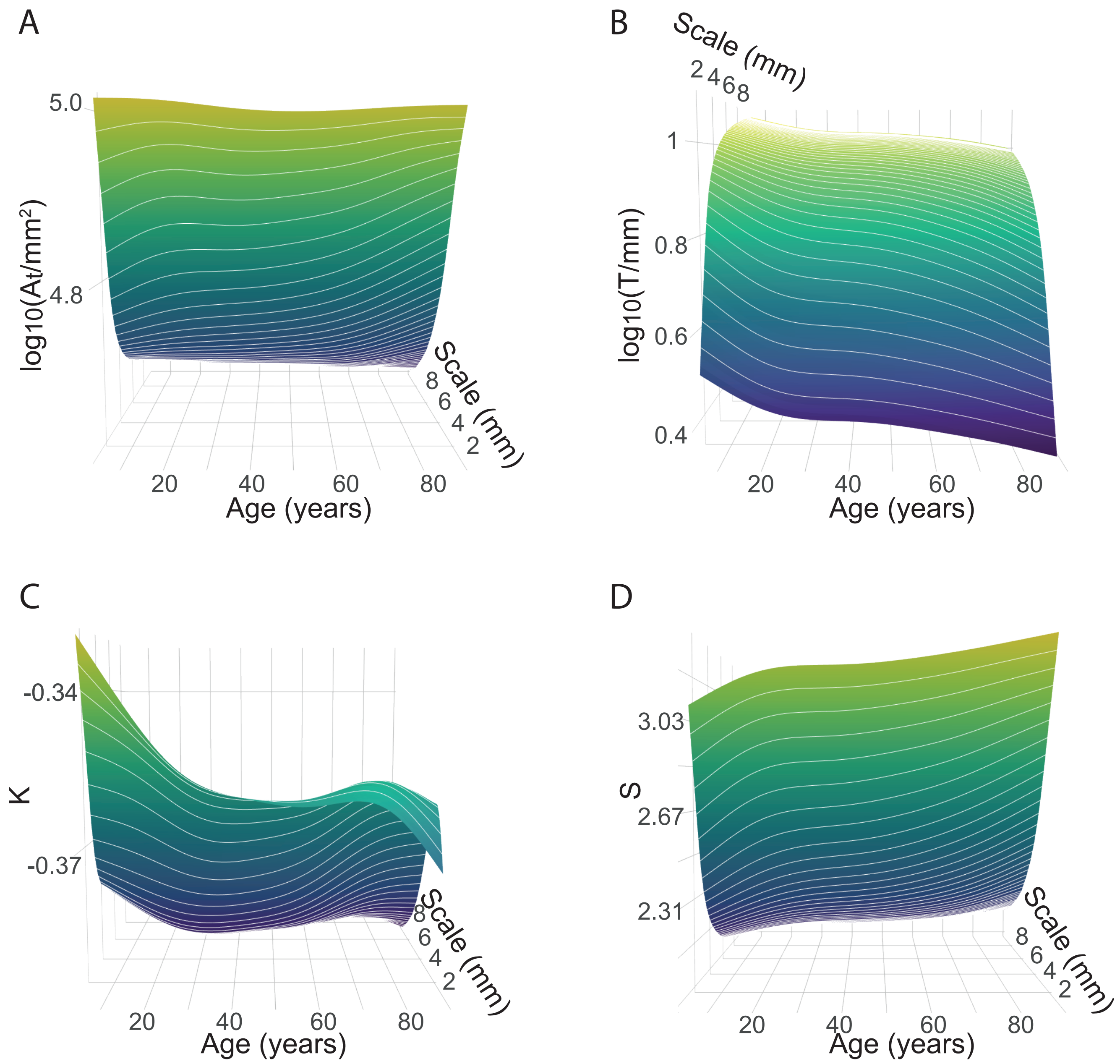

Figure S5.1: Lifespan trajectories in scales from 0.32 mm to 9 mm. A) Pial surface area $\log 10(A_t/mm^2)$. **B)** Average cortical thickness $\log 10(T/mm)$. **C)** Dimensionless metric K. **D)** Dimensionless metric S. The colour corresponds to the height of the plane, i.e. the value of the metric A_t, T, K, or S.

References

Barrick, T. R., Charlton, R. A., Clark, C. A., and Markus, H. S. (2010). White matter structural decline in normal ageing: A prospective longitudinal study using tract-based spatial statistics. *NeuroImage*, 51:565–577.

de Moraes, F. H., Mello, V. B., Tovar-Moll, F., and Mota, B. (2022). Establishing a Baseline for Human Cortical Folding Morphological Variables: A Multisite Study. *Frontiers in Neuroscience*, 16.

Ge, R., Yu, Y., Qi, Y. X., nan Fan, Y., Chen, S., Gao, C., Haas, S. S., New, F., Boomsma, D. I., Brodaty, H., Brouwer, R. M., Buckner, R., Caseras, X., Crivello, F., Crone, E. A., Erk, S., Fisher, S. E., Franke, B., Glahn, D. C., Dannlowski, U., Grotegerd, D., Gruber, O., Pol, H. E. H., Schumann, G., Tamnes, C. K., Walter, H., Wierenga, L. M., Jahanshad, N., Thompson, P. M., Frango, S., and Group, E. L. W. (2024). Normative modelling of brain morphometry across the lifespan with centilebrain: algorithm benchmarking and model optimisation. *The Lancet Digital Health*, 6:e211–e221.

Gunning-Dixon, F. M., Brickman, A. M., Cheng, J. C., and Alexopoulos, G. S. (2009). Aging of cerebral white matter: A review of mri findings. *International Journal of Geriatric Psychiatry*, 24:109–117.

Leiberg, K., Papasavvas, C., and Wang, Y. (2021). Local Morphological Measures Confirm that Folding Within Small Partitions of the Human Cortex Follows Universal Scaling Law. *Medical Image Computing and Computer Assisted Intervention – MICCAI 2021*, 12907 LNCS:691–700.

Mota, B. and Herculano-Houzel, S. (2015). Cortical folding scales universally with surface area and thickness, not number of neurons. *Science*, 349(6243):74–77.

Schilling, K. G., Archer, D., Rheault, F., Lyu, I., Huo, Y., Cai, L. Y., Bunge, S. A., Weiner, K. S., Gore, J. C., Anderson, A. W., and Landman, B. A. (2023a). Superficial white matter across development, young adulthood, and aging: volume, thickness, and relationship with cortical features. *Brain Structure and Function*, 228(3-4):1019–1031.

Schilling, K. G., Archer, D., Yeh, F.-C., Rheault, F., Cai, L. Y., Shafer, A., Resnick, S. M., Hohman, T., Jefferson, A., Anderson, A. W., Kang, H., and Landman, B. A. (2023b). Short superficial white matter and aging: A longitudinal multi-site study of 1293 subjects and 2711 sessions. *Aging Brain*, 3:100067.

Wang, Y., Leiberg, K., Ludwig, T., Little, B., Necus, J. H., Winston, G., Vos, S. B., de Tisi, J., Duncan, J. S., Taylor, P. N., and Mota, B. (2021). Independent components of human brain morphology. *NeuroImage*, 226:117546.

Wang, Y., Necus, J., Kaiser, M., and Mota, B. (2016). Universality in human cortical folding in health and disease. *Proceedings of the National Academy of Sciences of the United States of America*, 113(45):12820–12825.

Wang, Y., Necus, J., Rodriguez, L. P., Taylor, P. N., and Mota, B. (2019). Human cortical folding across regions within individual brains follows universal scaling law. *Communications Biology*, 2:191.
